# Use of the Hayami diffusive wave equation to model the relationship infected–recoveries–deaths of Covid-19 pandemic

**DOI:** 10.1017/S0950268821001011

**Published:** 2021-04-29

**Authors:** Roger Moussa, Samer Majdalani

**Affiliations:** 1 LISAH, Univ. Montpellier, INRAE, IRD, Montpellier SupAgro, Montpellier, France; 2 HSM, CNRS, IRD, Univ. Montpellier, Montpellier, France

**Keywords:** Coronavirus Covid-19, diffusive wave equation, epidemiology, Hayami kernel function, hydraulic, hydrology, *SIRD* compartmental models, unit hydrograph

## Abstract

Susceptible *S*-Infected *I*-Recovered *R*-Death *D* (*SIRD*) compartmental models are often used for modelling of infectious diseases. On the basis of the analogy between *SIRD* and compartmental models in hydrology, this study makes mathematical formulations developed in hydrology available for modelling in epidemiology. We adapt the Hayami model solution of the diffusive wave equation generally used in hydrological modelling to compartmental *I*–*R*–*D* models in epidemiology by simulating the relationships between the number of infectious *I*(*t*), the number of recoveries *R*(*t*) and the number of deaths *D*(*t*). The Hayami model is easy-to-use, robust and parsimonious. We compare the empirical one-parameter exponential model usually used in *SIRD* models to the two-parameter Hayami model. Applications were implemented on the recent Covid-19 pandemic. The application on data from 24 countries shows that both models give comparable performances for modelling the *I*–*D* relationship. However, for modelling the *I*–*R* relationship and the active cases, the exponential model gives fair performances whereas the Hayami model substantially improves the model performances. The Hayami model also presents the advantage that its parameters can be easily estimated from the analysis of the data distributions of *I*(*t*), *R*(*t*) and *D*(*t*). The Hayami model is parsimonious with only two parameters which are useful to compare the temporal evolution of recoveries and deaths in different countries based on different contamination rates and recoveries strategies. This study highlights the interest of knowledge transfer between different scientific disciplines in order to model different processes.

## Introduction

Since the pioneer study of [1] in epidemiology, *SIRD* (Susceptible *S*-Infected *I*-Recovered *R*-Death *D*) compartmental models are often used for modelling of infectious diseases, and are now used for the recent Covid-19 pandemic in China [2–4]. *SIRD* models can be used at the patch scale or over a large area [5–10], and can be coupled to probabilistic approaches [11, 12] and various processes such as population mobility [13–16]. When used at the scale of a patch, or of a country when the latter is considered as one homogeneous patch, *SIRD* models can be viewed similar to lumped hydrological models representing the links between several water cycle compartments [17] with rain as input (equivalent to the input *S*) and river flow, infiltration and evaporation as outputs (equivalent to the outputs *R* and *D*). Different hydrological modelling approaches were developed on the basis of an interaction between two categories of ‘functions’ called ‘production function’ and ‘transfer function’ [17, 18]. The production function separates the rain into runoff, infiltration and evaporation, and therefore distributes the mass balance between different compartments. Based on mass conservation, the transfer function is considered as a filter which transforms an input signal into an output signal, and calculates the time distribution of the production function outputs. In epidemiology, we can define by analogy a ‘production function’ to represent the relationship *S*–*I* which calculates the part of *S* contributing to *I*, and a ‘transfer function’ to represent the relationships *I*–*R*–*D*.

For the Covid-19 pandemic, the daily number of infected cases *I*(*t*), recoveries *R*(*t*) and deaths *D*(*t*) are freely available on websites of governmental institutions [19–21]. From graphics, we observe that the relationships *I*–*R* and *I*–*D* have similar shapes to those obtained from hydrologic transfer functions. First, mass conservation is verified because the total number of *I*(*t*) is equal to the total numbers of *R*(*t*) and *D*(*t*). Second, the output signals *R*(*t*) and *D*(*t*) can be derived from the input signal *I*(*t*) using a mathematical formulation identical to the transfer function describing the physical advection-dispersion processes: a lag time translation (for the advection process) and an attenuation of the peak (for the dispersion process). Although on the basis of analogical properties, transfer functions have been successfully used in other disciplines such as astronomy, geophysics, soil mechanics, meteorology, oceanography, traffic simulation or biological flows [22, 23], to our knowledge, no applications of the transfer functions were conducted for modelling *I*–*R*–*D* relationships in compartmental models in epidemiology.

This study aims to adapt transfer functions used in hydrology for modelling *I*–*R*–*D* relationships in epidemiology. *I*–*R*–*D* models are generally described by partial differential equations [24–26] where parsimonious first-order kinetics remains to be largely used because of their simplicity, robustness and their low number of parameters [27, 28]. In hydrology, under the hypotheses that the system is linear and time invariant, simplified versions of the transfer function were developed like the ‘unit hydrograph’, that is equivalent to a convolution with a kernel function representing the response to a Dirac input [29]. Furthermore, a linear reservoir is a one-parameter kernel function (or unit hydrograph) with an exponential decrease equivalent to first-order kinetics used in epidemiology (d*R*/d*t* = *γI* or d*D*/d*t* = *δI* with *γ* and *δ* as parameters). However, one-parameter unit hydrograph is not adapted to represent processes such as flood routing in channels where at least two-parameter unit hydrographs are needed to describe both advection and dispersion processes.

In epidemiology, first-order kinetics remains to be the simplest model [2, 16], and a question arises whether one-parameter first-order kinetics is sufficient to model at the country scale the *I*–*R*–*D* relationships for Covid-19, and whether simulations could be improved by using other mathematical formulations such as the two-parameter kernel functions. Various unit hydrographs have been developed [29], but one is of interest for possible applications in epidemiology: the Hayami model [30] used as a unit hydrograph resolution of the diffusive wave equation with parameters having a physical interpretation [31–34]. For applications in epidemiology, questions arise on the adaptability of the Hayami model to simulate the relationships *I*–*R*–*D*, the interpretation of model parameters and how could these parameters be used to compare different case studies.

This study attempts to make the Hayami solution of the diffusive wave equation available for modelling the *I*–*R*–*D* relationships in epidemiology. Applications were implemented on the actual 2020 Covid-19 pandemic. First, we present the *I*–*R*–*D* model, the Hayami model, its adaptation for compartmental models in epidemiology, and an application example to simulate *R* and *D* (given *I*) in the case of China where the pandemic is ending. Then, applications were implemented on data from 24 countries all round the world, with different levels of evolution of the pandemic, contamination rates and recoveries strategies. Finally, we show how the model parameters can be used to compare recoveries and deaths in different countries, and discuss the usefulness and the limitations of the model. The Supplementary material presents the theory of the unit hydrograph, and shows a comparison between the observed and calculated *R*(*t*), *D*(*t*) an *A*(*t*) for 24 countries.

## The *I*–*R*–*D* model

The *I*–*R*–*D* model developed herein is based on the unit hydrograph theory in hydrology adapted for applications in epidemiology. Inputs are the observed number of daily infected cases *I*_o_(*t*) and the observed mortality ratio *μ*, and outputs are the calculated daily recovered cases *R*_c_(*t*) and the calculated daily death cases *D*_c_(*t*). The performances of the *I*–*R*–*D* model are obtained by comparing the observed daily recovered cases *R*_o_(*t*) to the calculated *R*_c_(*t*), and the observed daily death cases *D*_o_(*t*) to the calculated *D*_c_(*t*).

**Fig. 1.**
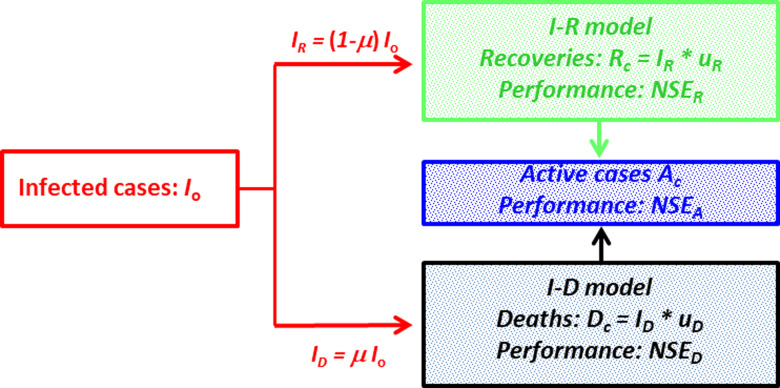
*I*–*R*–*D* model structure. The input *I*_o_(*t*) is the observed number of daily infected cases divided into *I_R_*(*t*) and *I_D_*(*t*) proportional to the mortality ratio *μ*. *t* is the time expressed in days. The *I*–*R* model calculates the daily number of recoveries *R*_c_(*t*), with a performance *NSE_R_*. The *I*–*D* model calculates the daily number of deaths *D*_c_(*t*), with a performance *NSE_D_*. The active cases *A*_c_(*t*) are calculated with a performance *NSE_A_*.

The distribution *I*_o_(*t*) is separated into two series: *I_R_*(*t*) representing the part of *I*_o_(*t*) that would recover, and *I_D_*(*t*) the part of *I*_o_(*t*) that would die (Fig. 1). We suppose a simple proportionality relationship between *I*_o_(*t*) and each of *I_R_*(*t*) and *I_D_*(*t*) according to:
1
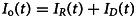

2
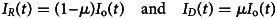

The *I*–*R*–*D* model is divided into two models: *I*–*R* and *I*–*D*. On the basis of the unit hydrograph theory [17, 29] (see details in the Supplementary material) we have
3
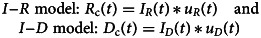

where the symbol ‘*’ represents the convolution relation, and *u_R_*(*t*) and *u_D_*(*t*) [*T*^−1^] are mathematical kernel functions with an integral equal to 1. For a Dirac (unit) input at *t* = 0, the output has the same mathematical equation as *u_R_*(*t*) (or *u_D_*(*t*)). In hydrology, the functions *u_R_*(*t*) and *u_D_*(*t*) are called unit hydrographs [29]. Section ‘Adapting the Hayami kernel function to epidemiology’ shows how these functions can be calculated.

The model also enables us to compare the observed cumulated number of recoveries *R*_to_(*t*) to the calculated *R*_tc_(*t*), the observed cumulated number of deaths *D*_to_(*t*) to the calculated *D*_tc_(*t*), and the observed daily active cases *A*_o_(*t*) to the calculated *A*_c_(*t*) with the below equation:
4
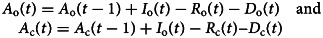


The performances of the *I*–*R* model, the *I*–*D* model and the active cases are calculated using the Nash–Sutcliffe efficiency (*NSE*) criteria [35] traditionally used in hydrology, with the below equations, respectively:
5
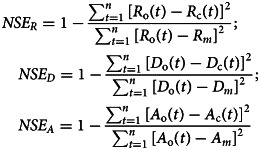

with *R_m_* the mean value of *R*_o_(*t*), *D_m_* the mean value of *D*_o_(*t*), *A_m_* the mean value of *A*_o_(*t*) and *n* the number of time steps. The criteria *NSE* is inferior to 1 with an optimum corresponding to *NSE* = 1. *NSE* = 0 corresponds to using the mean value of observations as a benchmark predictor and is regularly used as a benchmark to compare models [36, 37]. *NSE* < 0 indicates that the model is a worse predictor than the mean of observations. However the *NSE* is an indicator of performance which is sensitive to peak values. Increasingly an alternative metric, the Kling–Gupta efficiency (*KGE*) [38] is used instead with the below equations, respectively
6*a*
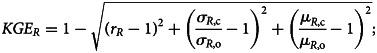

where *r_R_* is the linear correlation between *R*_o_(*t*) and *R*_c_(*t*), *σ_R_*_,o_ is the standard deviation of *R*_o_(*t*), *σ_R_*_,c_ is the standard deviation of *R*_c_(*t*), *μ_R_*_,o_ is the mean of *R*_o_(*t*) and *μ_R_*_,c_ is the mean of *R*_c_(*t*):
6*b*
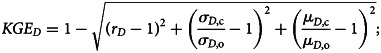

where *r_D_* is the linear correlation between *D*_o_(*t*) and *D*_c_(*t*), *σ_D_*_,o_ is the standard deviation of *D*_o_(*t*), *σ_D_*_,c_ is the standard deviation of *D*_c_(*t*), *μ_D_*_,o_ is the mean of *D*_o_(*t*) and *μ_D_*_,c_ is the mean of *D*_c_(*t*):
6*c*
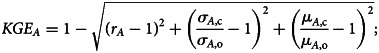

where *r_A_* is the linear correlation between *A*_o_(*t*) and *A*_c_(*t*), *σ_A_*_,__o_ is the standard deviation of *A*_o_(*t*), *σ_A_*_,__c_ is the standard deviation of *A*_c_(*t*), *μ_A_*_,__o_ is the mean of *A*_o_(*t*) and *μ_A_*_,__c_ is the mean of *A*_c_(*t*).

Knoben *et al*. [39] analysed the relationships between the *NSE* and the *KGE*, and concluded that *NSE* and *KGE* values cannot be directly compared because their relationship is non-unique. They also showed that using the mean observations as a benchmark does not result in *KGE* = 0 but *KGE* = −0.41. Thus *KGE* > −0.41 indicate that a model improves upon the mean observation benchmark.

In the following, we note the input *i*(*t*) for *I_R_*(*t*) or *I_D_*(*t*) and the output *o*(*t*) for *R*_c_(*t*) or *D*_c_(*t*). Although *i*(*t*) and *o*(*t*) are continuous functions in time in hydrological models, herein *i*(*t*) and *o*(*t*) are discrete functions representing the distribution of the number of cases per day, with *t* an integer representing the day number. In hydrology, different approaches can be used to calculate *u*(*t*) [29].

The exponential model, similar to the solution of the first-order kinetics used in compartmental models in epidemiology, is a unit hydrograph considered as a reference model as below:
7
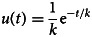

where *k* [*T*] is a parameter representing linear reservoir retention.

## Adapting the Hayami kernel function to epidemiology

**Fig. 2.**
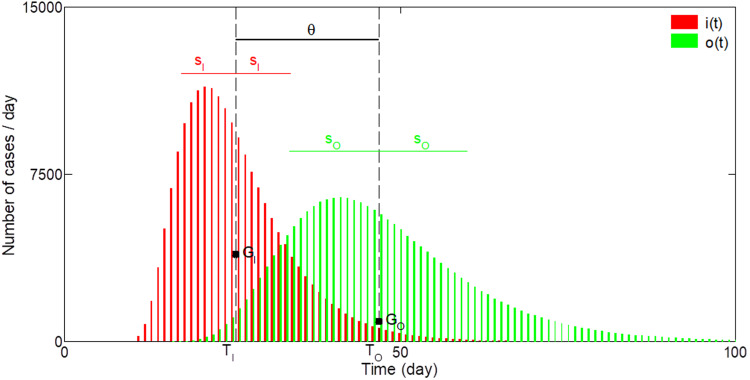
Example of the distributions of the input *i*(*t*) and the output *o*(*t*). *G_I_* and *G_O_* are, respectively, the centres of gravity of *i*(*t*) and *o*(*t*). *T_I_* and *T_O_* are the abscissae of *G_I_* and *G_O_* representing the means of *i*(*t*) and *o*(*t*). *s_I_* and *s_O_* are the standard deviations of *i*(*t*) and *o*(*t*). *θ* is the time delay between the two centres of gravity.

Saint-Venant [40] formulated the system of partial differential equations (continuity and momentum) to describe one-dimensional, gradually-varied, unsteady flow in rivers. The solution of the Saint-Venant equations has given rise to a number of numerical methods because no analytical solution is available. In practical applications of flood routing in natural channels, the acceleration terms in the Saint-Venant equations can be neglected, and the system is reduced to one parabolic equation, the diffusive wave equation [31, 32]:
8
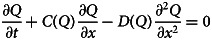

where *x* [*L*] is the length along the channel, *t* [*T*] is the time and the celerity *C*(*Q*) [*LT*^−1^] and the diffusivity *D*(*Q*) [*L*^2^*T*^−1^] are functions of the discharge *Q* [*L*^3^*T*^−1^]. Let *I*(*t*) and *O*(*t*) be, respectively, the upstream inflow and the downstream outflow. In the particular case of a semi-infinite channel, no physical downstream boundary condition exists, and *C*(*Q*) and *D*(*Q*) constant, the diffusive wave equation has as solution the analytical Hayami (1951) equation [30]:
9
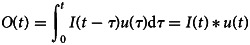

where *u*(*t*) is the Hayami kernel function:
10
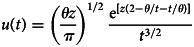

with two parameters *θ* [*T*] the lag time and *z* [dimensionless] a shape parameter. When *z* tends to zero, the Hayami equation has comparable shape as the exponential model equation (7). For hydraulic applications, equation (10) is generally written as a function of two physically based parameters, changing the two-parameter (*θ*, *z*) into (*C*, *D*), with *C* = *L/θ* (*L* being the length) and *D* = *L*^2^/(4*θz*). The use of the two-parameter (*C*, *D*) is more adapted for hydraulic application because both parameters have physical significance. The use of the two-parameter (*θ*, *z*) is more adapted for hydrologic applications on catchments because *θ* represents the lag time between *i*(*t*) and *o*(*t*), and can be thus estimated. However, *z* is empirical and needs to be calibrated. For applications in epidemiology, the parameter *θ* represents the lag time between either *I_R_*(*t*) and *R*_o_(*t*), or *I_D_*(*t*) and *D*_o_(*t*), and hence can be easily interpreted, whereas the parameter *z* has no significance for the relationships *I*–*R* and *I*–*D*. This is why equation (10) needs to be rewritten according to two parameters that can be more easily interpreted in epidemiology. For that, *i*(*t*) and *o*(*t*) can be considered as distributions characterised respectively as below (Fig. 2):

- *G_I_* and *G_O_*: the centres of gravity.
- *T_I_* and *T_O_*: the abscissae of *G_I_* and *G_O_* which represent the means of *i*(*t*) and *o*(*t*).
- *s_I_* and *s_O_*: the standard deviations of *i*(*t*) and *o*(*t*), and hence *s_I_*^2^ and *s_O_*^2^ are the variances.

The values of *T_I_* and *T_O_* depend on the time origin (*t* = 0) whereas *s_I_* and *s_O_* do not depend on time origin. Following Moussa [31, 32], we have
11
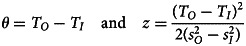


The parameter *θ* represents the time delay between the two centres of gravity *G_I_* and *G_O_*. Let *τ* be
12
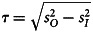


The new parameter *τ* has a time dimension, and is proportional to the square root of the difference of the variance of *o*(*t*) and *i*(*t*). Combining equations (11) and (12), we have the relationships:

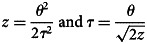


Substituting equation (13) in (10) we obtain the expression of the Hayami unit hydrograph function of (*θ*, *τ*) instead of (*θ*, *z*):
14
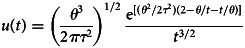


The Hayami *u*(*t*) given by equation (14) is applicable for the continuous function data. But, the Covid-19 pandemic problem dealing with *I*–*R*–*D* and active variables are observed in discrete time interval of 1 day. This necessitates the conversion of the continuous function *u*(*t*) to discrete 1-day time interval. To obtain at the discrete *u*(*t*) of the Hayami model at Δ*t* time interval (with Δ*t* = 1 day herein), we use the discrete *u*(*t*) of the Hayami model using a similar approach as the method described by [41] (see details in the Supplementary material). Another solution consists of subdividing the daily time step into finer numerical time steps under the hypothesis of uniform distribution of data. The finer the time step, the closer one gets to a continuous function of time (see the sensitivity analysis for Δ*t* = 10 min, 1 h, 3 h and 1 day in Section ‘Sensitivity analysis of the *I*–*R*–*D* Hayami model to the time step of calculation’). The *I*–*R*–*D* model proposed herein was developed in Matlab^®^ and integrated in the MHYDAS hydrological model (MHYDAS-IRD) [42].

The two parameters (*θ*, *τ*) are related to the means and variances of the two distributions *i*(*t*) and *o*(*t*) by equations (10) and (11). The advantage of equation (14) using (*θ*, *τ*) instead of (*θ*, *z*) is that both parameters (*θ*, *τ*) can be estimated from data analysis without any calibration. However, the calibration of (*θ*, *τ*) can be also undertaken in order to improve the model performances.

**Fig. 3.**
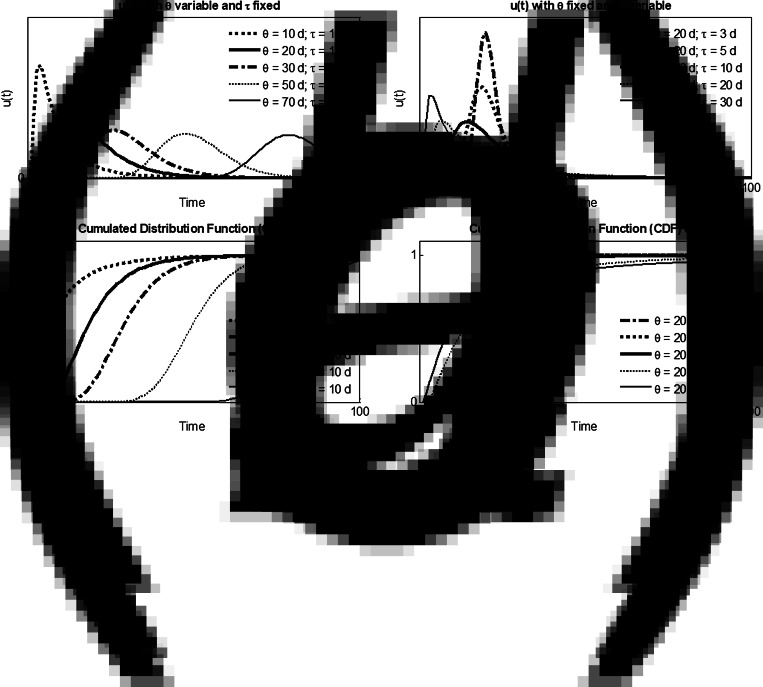
Examples of the Hayami unit hydrograph *u*(*t*). (a) For *τ* = 10 days and different values of *θ*. (b) For *θ* = 20 days and different values of *τ*. (c) CDF of (a) and (d) CDF of (b).

Figure 3a shows the shape of the output distribution for different values of *θ*. A small *θ* value gives a sharp output distribution with a small time delay between the input and the output, whereas a large *θ* value gives a damped output distribution with a large time delay between the input and the output. Figure 3b shows the output distribution for different values of *τ*. A small *τ* value gives a damped and more asymmetric output distribution with a small time delay between the input and the output, whereas a large *τ* value gives a sharp and more symmetric output distribution with a large time delay between the input and the output. Figures 3c and d show the cumulative distribution function (CDF) of Figures 3a and b: they represent the time evolution of the total number of *o*(*t*) when *i*(*t*) is a Dirac delta function.

In the applications, we compare the reference one-parameter exponential model (equation 7) with *k_R_* the parameter of *u_R_*(*t*) and *k_D_* the parameter of *u_D_*(*t*), to the two-parameter Hayami model (equation 14) with (*θ_R_*, *τ_R_*) the parameters of *u_R_*(*t*) and (*θ_D_*, *τ_D_*) the parameters of *u_D_*(*t*). The mortality ratio *μ* is supposed to be known from the observed data. For both models, the parameters of *u_R_*(*t*) are calculated separately from those of *u_D_*(*t*). The performances are *NSE_R_* and *KGE_R_* for *I*–*R*, *NSE_D_* and *KGE_D_* for *I*–*D* and *NSE_A_* and *KGE_A_* for the active cases (equations (5) and (6)). The parameters are calibrated using an iterative automatic trial-and-error method maximising the *NSE* criteria. This method can be used for both exponential and Hayami models, for datasets (*I*_o_(*t*), *R*_o_(*t*) and *D*_o_(*t*)) that are partial (pandemic is evolving) or complete (pandemic is ending).

## Applications on the Covid-19 pandemic

### Data

Applications were implemented on the actual Covid-19 pandemic which occurred in Wuhan, Hubei Province, China, in December 2019. Data are available from Worldometer [20] Covid-19 data used by Johns Hopkins CSSE [19], governmental institutions and many others [21]. Daily data for *I*_o_(*t*), *R*_o_(*t*) and *D*_o_(*t*) are available from 1 January until 19 May 2020 for 24 countries: Australia (denoted AU), Austria (AT), Belgium (BE), China (CN), Cuba (CU), Czechia (CZ), Denmark (DK), France (FR), Germany (DE), Iceland (IS), Iran (IR), Italy (IT), Japan (JP), Malaysia (MY), New Zealand (NZ), Romania (RO), Slovakia (SK), South Korea (KR), Spain (ES), Switzerland (CH), Thailand (TH), Turkey (TU), USA (US) and the whole world (World). The latter case enables us to study an overall average trend in the whole world.

**Fig. 4.**
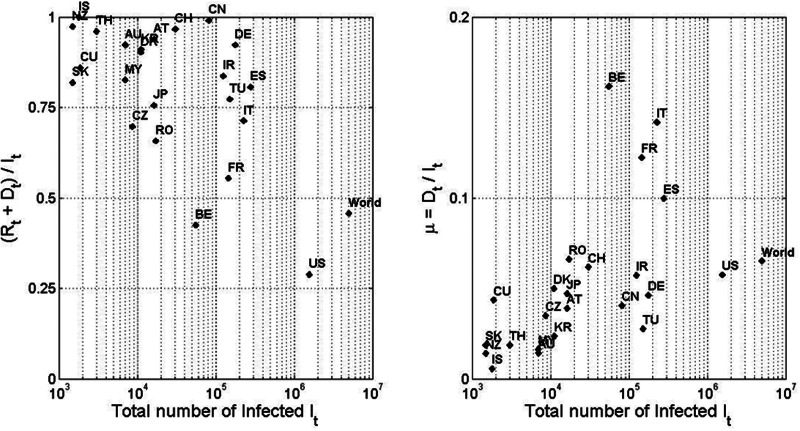
Data characteristics for the 24 countries, where for each country *I_t_* is the total number of infected cases, *R_t_* the total number of recoveries and *D_t_* the total number of deaths. Data are available from 1 January to 19 May 2020. (a) Pandemic evolution index (*R_t_* *+* *D_t_*)/*I_t_* function of *I_t_* and (b) mortality ratio: *μ* = *D_t_*/*I_t_*.

Figure 4 shows the characteristics of the data for all 24 countries, where for each country *I_t_* is the total number of infected cases, *R_t_* the total number of recoveries and *D_t_* the total number of deaths. Figure 4a shows the large range of variability of *I_t_* with 2 × 10^3^ (IS, NZ, SK) < *I_t_* < 5 × 10^6^ (World). We define also the index (*R_t_* + *D_t_*)/*I_t_* representing the evolution of the pandemic at the date of data availability 19 May 2020: the index is close to 1 when the pandemic is ending (e.g. CN, IS, NZ) and lowest than 0.5 (e.g. BE, US, World) when the pandemic is still actively evolving. Figure 4b shows the large range of variability of the mortality ratio *μ* = *D_t_*/*I_t_* with 0.005 (IS) < *μ* < 0.16 (BE). Probably different data collection strategies have been carried out among the different countries. However, the *I*–*R*–*D* model was applied separately to each country, and therefore the calibrated sets of parameters can be considered as descriptors of the *I*–*R* and *I*–*D* relationships for each country.

We compare the exponential and the Hayami *I*–*R*–*D* models to simulate the relationships *I*–*R*, *I*–*D* and the active cases. As we observe noisy signals for *I*_o_(*t*), *R*_o_(*t*) and *D*_o_(*t*), simulations can be improved by smoothing these signals. For both the exponential and the Hayami models, we also compare four smoothing strategies: without smoothing, 3-days moving average, 5-days moving average, and 7-days moving average. A sensitivity analysis was also undertaken to study the impact of the time step of calculation on the calibrated parameters and the model performances, by subdividing the daily time step into finer numerical time steps (Δ*t* = 10 min, 1 h, 3 h and 1 day). For the *I*–*R* model, we note *NSE_R_, NSE_R_*_3_, *NSE_R_*_5_ and *NSE_R_*_7_, the values of *NSE* respectively for the four smoothing strategies. For the *I*–*D* model, we note *NSE_D_*, *NSE_D_*_3_, *NSE_D_*_5_ and *NSE_D_*_7_. For the active cases, we note *NSE_A_, NSE_A_*_3_, *NSE_A_*_5_ and *NSE_A_*_7_. For the *I*–*R* model, we note *KGE_R_*, *KGE_R_*_3_, *KGE_R_*_5_ and *KGE_R_*_7_, the values of *KGE* respectively for the four smoothing strategies. For the *I*–*D* model, we note *KGE_D_*, *KGE_D_*_3_, *KGE_D_*_5_ and *KGE_D_*_7_. For the active cases, we note *KGE_A_*, *KGE_A_*_3_, *KGE_A_*_5_ and *KGE_A_*_7_.

First, we present an example of application and the sensitivity analysis on China where the pandemic is ending. Then, we show the results for all 24 countries, for both the exponential and Hayami models, and for the three smoothing strategies. The Supplementary material shows the comparison between the observed {*R*_o_(*t*), *D*_o_(*t*), *A*_o_(*t*)} and the calculated {*R*_c_(*t*), *D*_c_(*t*), *A*_c_(*t*)} for all countries.

### Application on China

**Fig. 5.**
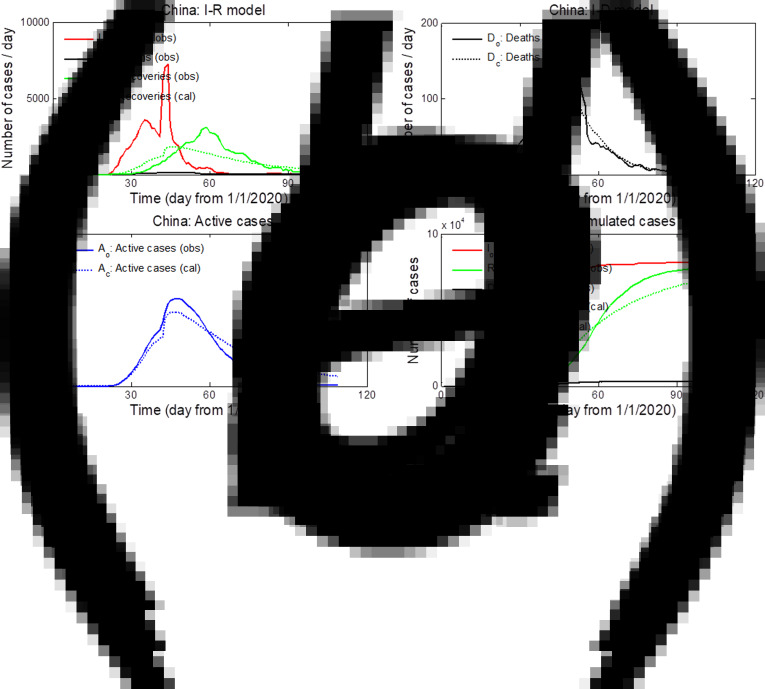
*I*–*R*–*D* exponential model application on Covid-19 in China (with 3-days smoothing average of data) using the calibrated parameters. (a) Comparison of the observed and calculated recoveries; (b) comparison of the observed and calculated deaths; (c) comparison of the observed and calculated active cases; (d) comparison of the cumulated observed infected cases, the observed and calculated recoveries, and the observed and calculated deaths. Data are available from 1 January 2020 to 19 May 2020.

**Fig. 6.**
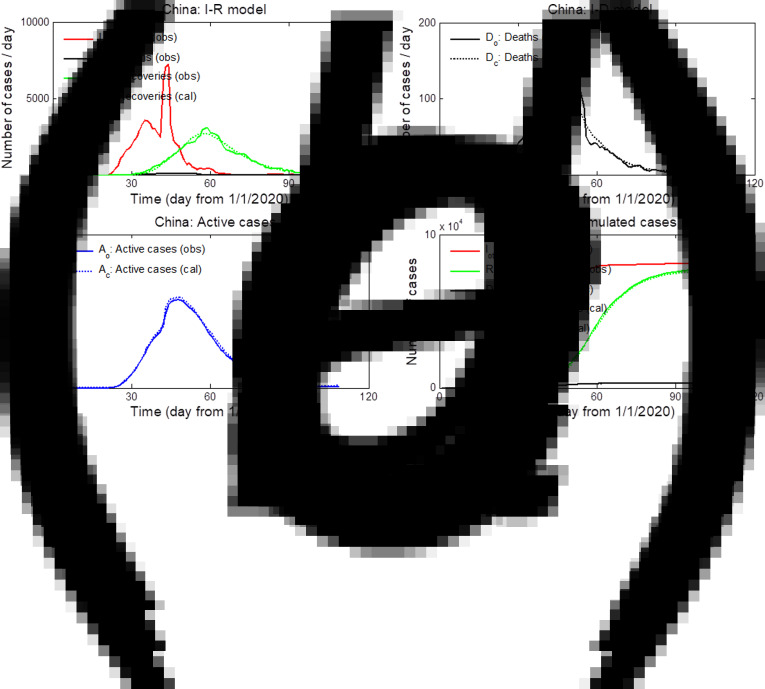
*I*–*R*–*D* Hayami model application on Covid-19 in China (with 3-days smoothing average of data) using the calibrated parameters. (a) Comparison of the observed and calculated recoveries; (b) comparison of the observed and calculated deaths; (c) comparison of the observed and calculated active cases; (d) comparison of the cumulated observed infected cases, the observed and calculated recoveries, and the observed and calculated deaths. Data are available from 1 January 2020 to 19 May 2020.

First, we study in detail the case of China where the pandemic was ending by the mid of April 2020. The total number of infected *I* is 82 123 cases, recoveries *R* is 78 042 cases and deaths *D* is 3324 cases and the mortality ratio is *μ* = 0.041. Figures 5 and 6 show the observed {*R*_o_(*t*), *D*_o_(*t*), *A*_o_(*t*)} and the calculated {*R*_c_(*t*), *D*_c_(*t*), *A*_c_(*t*)} for respectively the exponential and the Hayami models after calibration of the parameters using data with 3-days moving average. For the *I*–*R* model, the calibrated parameters of *u_R_*(*t*) are for the exponential model *k_R_* = 26.5 days with fair performance *NSE_R_*_3_ = 0.648 (Fig. 5a), and for the Hayami model *θ_R_* = 21.8 days and *τ_R_* = 10.3 days (very close to those calculated from equation (10) *θ_R_* = 20.3 days and *τ_R_* = 9.5) with excellent performance *NSE_R_*_3_ = 0.99 (Fig. 6a). The calibrated values of the lag time *k_r_* and *θ_R_* are comparable to cure rate for infections (17–20 days) obtained in [4]. For the *I*–*D* model, the calibrated parameters of *u_D_*(*t*) are for the exponential model *k_D_* = 9.3 days with very good performance *NSE_D_*_3_ = 0.93 (Fig. 5b), and for the Hayami model *θ_D_* = 10.1 days and *τ_D_* = 11.1 days with excellent performance *NSE_D_*_3_ = 0.95 (Fig. 6b). When comparing the observed and calculated active cases *A*(*t*), we observe that the exponential model gives good results *NSE_A_*_3_ = 0.82 (Fig. 5c) whereas the Hayami model gives much better results *NSE_A_*_3_ = 0.95 (Fig. 6c). Finally, when comparing the cumulated observed and calculated number of recoveries, and the cumulated observed and calculated number of deaths, we observe also fair performance of the exponential model (Fig. 5d) and excellent performance of the Hayami model (Fig. 6d).

**Fig. 7.**
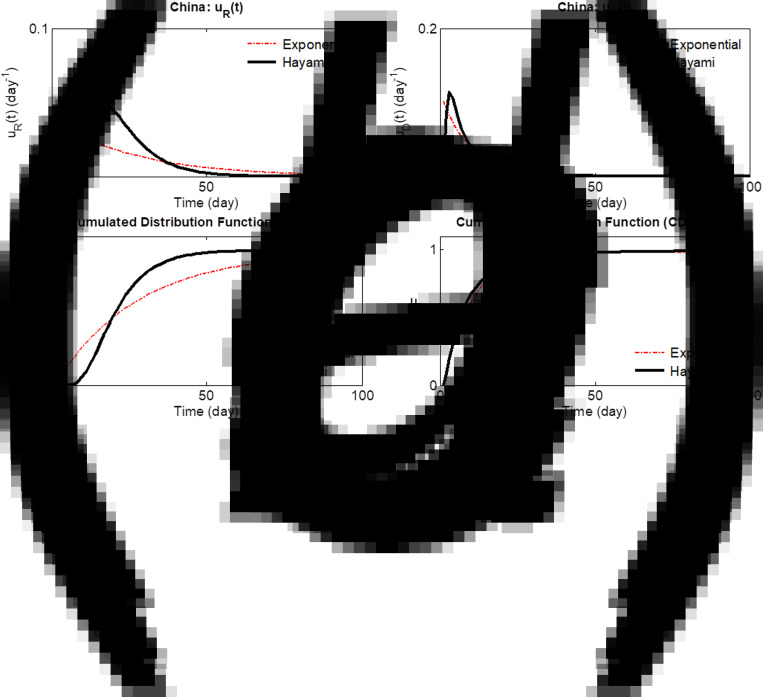
Comparison of the unit hydrographs calibrated for the exponential and Hayami models for the Covid-19 in China: (a) *u_R_*(*t*); (b) *u_D_*(*t*); (c) CDF of (a) and (d) CDF of (b).

These results show that the one-parameter exponential model is sufficient and give comparable results to the Hayami model only for modelling the relationship *I*–*D*. However, for modelling the relationship *I*–*R* and the active cases, the Hayami model highly improves the performances in comparison with the exponential model, presenting also the advantage that its parameters can be easily estimated from the analysis of the data distributions of *I*(*t*) and *R*(*t*). Figure 7 shows the *u_R_*(*t*) and *u_D_*(*t*) obtained with the calibrated parameters for China. For *u_R_*(*t*) (Fig. 7a and the corresponding CDF in Fig. 7c), we observe different behaviours for the two models. The exponential model cannot reproduce a unit hydrograph that rises to a maximum and then falls down, which explains the fair performance of the exponential model. However, the Hayami model succeeds because it enables us to take into account a lag time translation (comparable to the advection process) and an attenuation of the peak (comparable to the dispersion process). For *u_D_*(*t*) (Fig. 7b and the corresponding CDF in Fig. 7d), we observe very comparable results for both models.

### Sensitivity analysis of the *I*–*R*–*D* Hayami model to the time step of calculation

This section presents a sensitivity analysis of the Hayami *I*–*R*–*D* model on China. Compared to applications in hydrology where the discharge is a continuous function of time, the major difference for epidemiological applications of the Hayami *I*–*R*–*D* model is that in epidemiology the data are a discrete time function representing the daily number of cases. However, all unit hydrograph approaches, including the Hayami model, can be easily adapted for applications on discrete or continuous data as discussed in Section ‘Adapting the Hayami kernel function to epidemiology’ [41, 43]. Around the reference data time step Δ*t* = 1 day, we conduct a sensitivity analysis by subdividing the daily time step into finer time steps (Δ*t* = 10 min, 1 h and 3 h) under the assumption of a uniform distribution of the number of cases. The finer the time step Δ*t*, the closer one gets to a continuous function of time. For the data used, we also compare four smoothing strategies: without smoothing, 3-days moving average, 5-days moving average and 7-days moving average.

Table 1 shows the values of the calibrated parameters of the *I*–*R* model (*θ_R_* and *τ_R_*) and the corresponding criteria functions (*NSE_R_* and *KGE_R_*), the calibrated parameters of the *I*–*D* model (*θ_D_* and *τ_D_*) and the corresponding criteria functions (*NSE_D_* and *KGE_D_*), and the criteria functions corresponding to the actives cases (*NSE_A_* and *KGE_A_*). For the fine time steps (10 min, 1 h and 3 h), the calibrated parameters and the corresponding performance criteria remain very close in comparison with those obtained with the reference time step of 1 day. This is due to the fact that the use of a fine time step only allows tending to continuous time functions (i.e. Δ*t* = 10 min) but using the same data as for Δ*t* = 1 day. However, we observe an improvement in model performances and a slight change in the values of the set parameters when the original data are smoothed (21.5 < *θ_R_* < 21.8 days and 10.3 <  *τ_R_* < 10.5 days). The improvement of model performances comparing the data without smoothing to 7-days moving average smoothed data are as follows: *NSE_R_* = 0.966 and 0.992, *KGE_R_* = 0.603 and 0.619, *NSE_D_* = 0.915 and 0.978, *KGE_R_* = 0.966 and 0.987, *NSE_A_* = 0.997 and 0.998, *KGE_A_* = 0.739 and 0.740. As the model results are more sensitive to smoothing data then to the use of finer time steps, in the following we limit the applications to compare the performance of the models using the daily time step for the four smoothing strategies (without smoothing, 3-days, 5-days moving and 7-days moving average).

**Table 1.** *I*–*R*–*D* Hayami model application on Covid-19 in China for different time steps analysis by subdividing the daily time step into finer time steps (Δ*t* = 10 min, 1 h, 3 h and 1 day) and four smoothing strategies (without smoothing, 3-days moving average, 5-days moving average and 7-days moving average): the calibrated parameters of the *I–R* model (*θ*_*R*_ and *τ_R_*) and the corresponding criteria functions (*NSE_R_* and *KGE_R_*), the calibrated parameters of the *I–D* model (*θ*_*D*_ and *τ_D_*) and the corresponding criteria functions (*NSE_D_* and *KGE_D_*), and the criteria functions corresponding to the actives cases (*NSE_A_* and *KGE_A_*).

	*I*–*R* model				*I*–*D* model				Active cases	
Δ*t*	*θ_R_* (days)	*τ_R_* (days)	*NSE_R_*	*KGE_R_*	*θ_D_* (days)	*τ_D_* (days)	*NSE_D_*	*KGE_D_*	*NSE_A_*	*KGE_A_*
	Without smoothing									
10 min	21.6	10.5	0.973	0.606	10.2	11.0	0.919	0.967	0.997	0.739
1 h	21.6	10.5	0.971	0.604	10.2	11.0	0.918	0.967	0.997	0.739
3 h	21.7	10.4	0.969	0.604	10.1	11.0	0.918	0.967	0.997	0.739
1 day	21.8	10.3	0.966	0.603	10.1	11.1	0.915	0.966	0.997	0.739
	Smoothing using 3-days moving average									
10 min	21.5	10.5	0.986	0.614	10.3	11.0	0.956	0.982	0.997	0.739
1 h	21.6	10.5	0.986	0.614	10.2	11.0	0.955	0.982	0.997	0.739
3 h	21.6	10.4	0.985	0.612	10.2	11.1	0.953	0.982	0.997	0.739
1 day	21.7	10.3	0.984	0.611	10.2	11.1	0.953	0.982	0.997	0.739
	Smoothing using 5-days moving average									
10 min	21.5	10.5	0.990	0.618	10.3	11.1	0.971	0.988	0.998	0.740
1 h	21.5	10.5	0.990	0.617	10.3	111	0.970	0.987	0.998	0.740
3 h	21.6	10.4	0.989	0.615	10.2	11.2	0.969	0.986	0.998	0.740
1 day	21.6	10.4	0.988	0.615	10.2	11.2	0.968	0.985	0.998	0.740
	Smoothing using 7-days moving average									
10 min	21.5	10.5	0.993	0.621	10.2	11.1	0.980	0.988	0.998	0.740
1 h	21.6	10.5	0.992	0.619	10.2	11.2	0.980	0.988	0.998	0.740
3 h	21.6	10.5	0.992	0.619	10.3	11.2	0.979	0.987	0.998	0.740
1 day	21.6	10.4	0.992	0.619	10.3	11.2	0.978	0.987	0.998	0.740

### Application on 24 countries

This section aims to show how the methodology can be applied, and how the parameters could be used to analyse the variability among different countries. For a part of the 24 countries, the pandemic is still evolving, and the series *I*_o_(*t*), *R*_o_(*t*) and *D*_o_(*t*) are still incomplete. Hence, the parameters of both the exponential and the Hayami models calibrated on the available data, may slightly change when the pandemic ends. For each country, we obtain generally similar values of the calibrated parameters for the four smoothing strategies (without smoothing, 3-days moving average, 5-days moving average and 7-days moving average) but the performances (*NSE* and *KGE*) may vary drastically with the smoothing strategy.

**Fig. 8.**
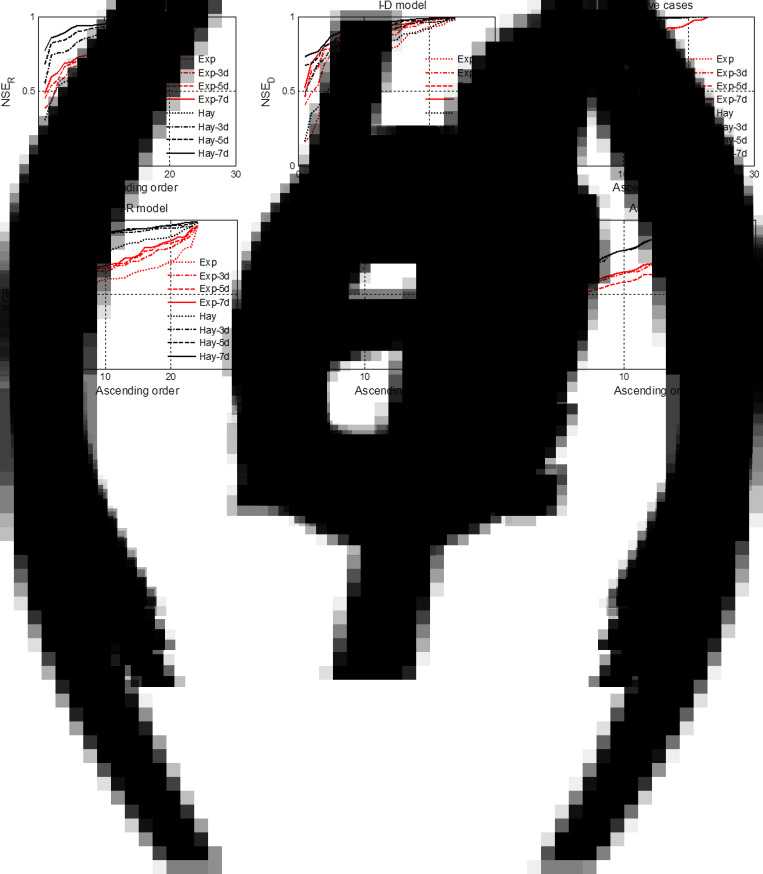
For the *I*–*R* model (a and d), the *I*–*D* model (b and e) and the active cases (c and f), comparison of the exponential model (denoted Exp) and the Hayami model (Hay) for four different smoothing strategies: without smoothing, 3-days moving average (3d), 5-days moving average (5d), and 7-days moving average (7d). The values of the *NSE* (a, b and c) and *KGE* (d, e and f) performance criteria are classified by ascending order for the 24 countries.

**Fig. 9.**
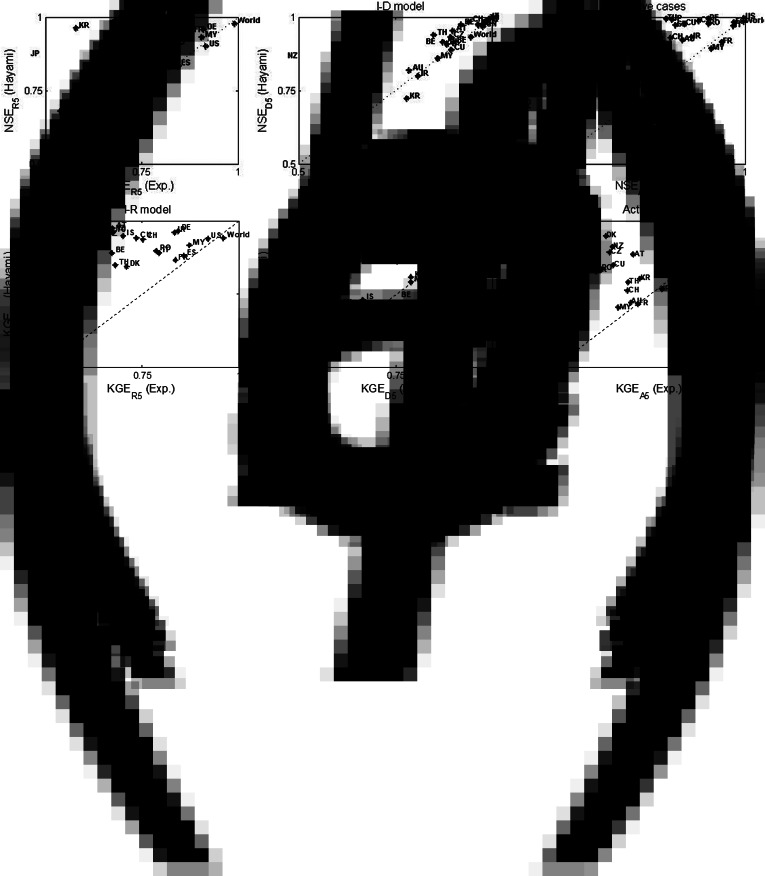
Comparison of the performances *NSE* (a, b and c) and *KGE* (d, e and f) of the exponential (denoted Exp) and the Hayami models (smoothing data with 5-days moving average) for : the *I*–*R* model (a and d), the *I*–*D* model (b and e), and the active cases (c and f).

Figure 8 shows a comparison of the *NSE* (Figs 8a–c) and *KGE* (Figs 8d–f) of the exponential and the Hayami models for the four smoothing strategies for all studied countries. For the *I*–*R* model, the 7-days moving average improves the performances *NSE* (Fig. 8a) of both the exponential and the Hayami models in comparison with the two remaining smoothing strategies. Moreover, the Hayami model performs much better than the exponential model: for the 5-days moving average, *NSE_R_*_5_ > 0.90 for four countries with the exponential model, and for 20 countries with the Hayami model (Fig. 9a). For the *I*–*D* model, Figure 8b shows that for both the exponential and the Hayami models, the 3-days, 5-days and 7-days moving average give similar results but better than without smoothing. The Hayami model gives comparable and slightly better results than the exponential model: for the 5-days moving average, *NSE_D_*_5_ > 0.90 for 12 countries with the exponential model, and for 16 countries with the Hayami model (Fig. 9b). For the active cases, Figure 8c shows that the three smoothing strategies give similar results for both the exponential and the Hayami models, because the active cases is a cumulated function, and consequently is less sensitive to noisy data. However, the Hayami model gives better results than the exponential model: for the 5-days moving average, *NSE_A_*_5_ > 0.95 for nine countries with the exponential model, and for all 24 countries with the Hayami model (Fig. 9c).

Similar results are obtained with the *KGE*. The Hayami model performs much better than the exponential model: for the 5-days moving average, *KGE_R_*_5_ > 0.90 for two countries with the exponential model, and for 16 countries with the Hayami model (Fig. 9d); *KGE_D_*_5_ > 0.90 for six countries with the exponential model, and for 12 countries with the Hayami model (Fig. 9e); *KGE_A_*_5_ > 0.90 for seven countries with the exponential model, and for 14 countries with the Hayami model (Fig. 9e).

**Fig. 10.**
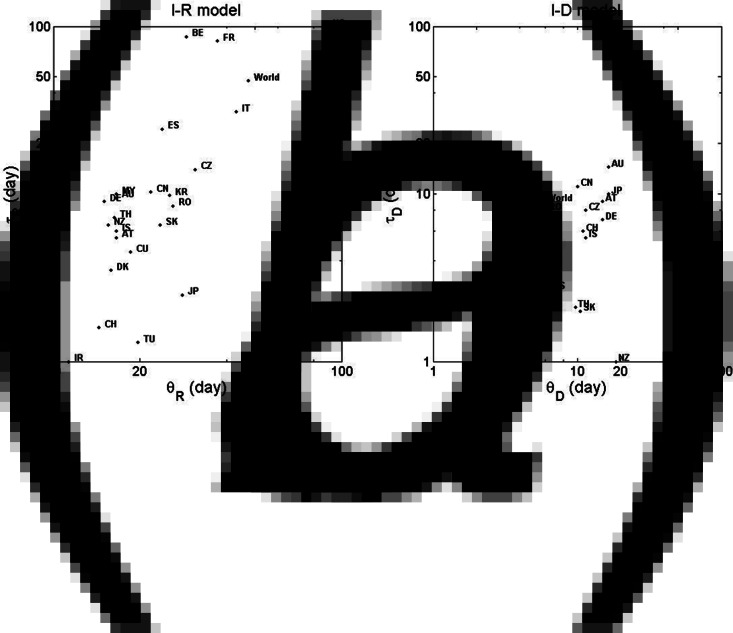
Hayami calibrated parameters for the 24 countries for: (a) *I*–*R* model (*θ_R_* and *τ_R_*) and (b) *I*–*D* model (*θ_D_* and *τ_D_*).

Finally, Figure 10 shows the Hayami calibrated parameters for the 24 countries for both the *I*–*R* model (*θ_R_* and *τ_R_*) and the *I*–*D* model (*θ_D_* and *τ_D_*). The parameters range are: 11 days (IR) < *θ_R_* < 89 days (US), 1.5 days (IR) < *τ_R_* < 99 days (US), 4 days (DK, MY) < *θ_D_* < 30 days (KR), 1 day (NZ)  < *τ_D_* < 27 days (KR). High values of *θ_R_* (or *θ_D_*) correspond to a long time interval between infection and recoveries (or deaths). High values of *τ_R_* (or *τ_D_*) correspond to a high difference of the variance between *I*_o_(*t*) and *R*_o_(*t*) (or *I*_o_(*t*) and *D*_o_(*t*)) as given in equation (9) and shown in Figure 2. Some particular cases can be examined such as Iran which has the shortest recovery period of *θ_R_* = 11 days with small *τ_R_* = 1 day in comparison with results of other countries. A small value of *θ_R_* can be due either to a late date of detection of infected cases and consequently a shorter recovery period, or to the recovery criterion with, for example a single negative test instead of two for recovered patients, which shortens the contamination period. The small value of *τ_R_* is an indicator that both *I*(*t*) and *R*(*t*) have the same dynamic with low diffusion. Conversely, France and the US data are characterised by a large *τ_R_* (respectively, 82 and 99). This is mainly due to the fact that the *R*(*t*) curves were rising and incomplete by mid-May 2020. Consequently, the calibration of the *I*–*R*–*D* model on the *NSE* criterion favouring the high values of the observation can induce a poor estimation of the parameters. This is not the case where the pandemic is over (e.g. Austria, China, Germany, Iceland, New Zealand, etc.), where small values of *τ_R_* (between 6 and 10 days) are calibrated.

The four parameters (*θ_R_*, *τ_R_*, *θ_D_* and *τ_D_*) are descriptors of the relationships between the distributions *I*(*t*), *R*(*t*) and *D*(*t*). Consequently, they depend on various factors such as the measurement strategy of each country, the health policy, the population density, the presence and date of closure of the local airport, etc. Given the heterogeneities of countries, and the time evolution of health policies, it could be hard to explain as well. However, these parameters remain useful indicators on data acquisition, recoveries strategies and pandemic evolution. They are also useful to compare and classify countries and regions.

**Fig. 11.**
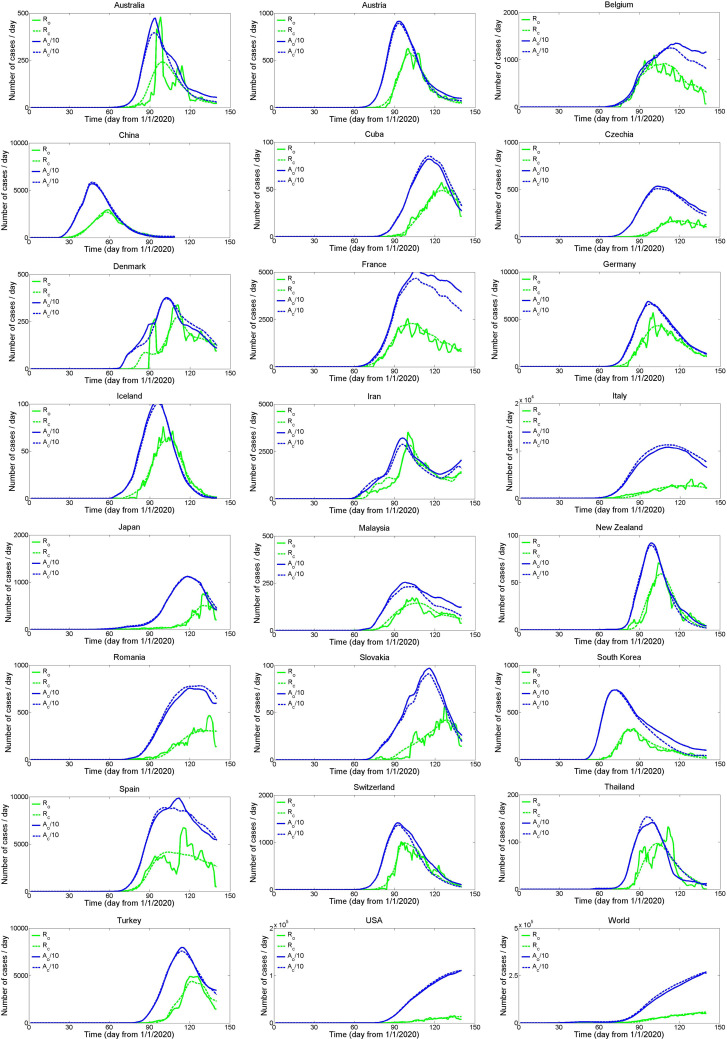
Comparison between the observed *R*_o_(*t*) and the calculated *R*_c_(*t*) recoveries, and between the observed *A*_o_(*t*) and the calculated *A*_c_(*t*) active cases, for 24 countries using the Hayami *I*–*R*–*D* model (smoothing data with 5-days moving average).

Finally, Figure 11 shows the overall very good performances of the *I*–*R*–*D* Hayami model (with 5-days moving average data smoothing) for the 24 countries, comparing the observed *R*_o_(*t*) and the calculated *R*_c_(*t*) recoveries, and the observed *A*_o_(*t*) and the calculated *A*_c_(*t*) active cases. Calibrated parameters and model performances will probably change when additional data will be available. However, these applications should be seen as first tests that show that the Hayami solution of the diffusive wave equation, and more generally the unit hydrograph theory, can be easily adapted to compartmental *I*–*R*–*D* models in epidemiology. In hydrology, the input *i*(*t*) and the output *o*(*t*) signals are continuous functions, whereas *I*_o_(*t*), *R*_o_(*t*) and *D*_o_(*t*) functions in epidemiology are discrete functions. Despite discontinuities in data, and the different methods among countries to define the observed *I*_o_(*t*), *R*_o_(*t*) and *D*_o_(*t*), the *I*–*R*–*D* model provides simulations mostly qualified as excellent when slightly smoothing the noisy data. The Hayami model is easy-to-use and parsimonious with only two parameters for each of *u_R_*(*t*) and *u_D_*(*t*). One main advantage of the Hayami model is that both parameters (*θ*, *t*) can be estimated using equation (11) from the analysis of *I*_o_(*t*), *R*_o_(*t*) and *D*_o_(*t*) when the pandemic ends, avoiding the calibration procedure. The parameters, either calibrated or estimated, are useful simple describers to compare the temporal evolution of recoveries and deaths in different countries.

## Conclusion

On the basis of the analogy between *SIRD* and compartmental models in hydrology, this study makes mathematical formulations developed in hydrology available for modelling in epidemiology. We adapt the ‘transfer functions’ generally used in hydrological modelling to compartmental *I*–*R*–*D* models in epidemiology in order to simulate the relationships between the number of infectious *I*(*t*), the number of recovered *R*(*t*), the number of death cases *D*(*t*) and the number of active cases *A*(*t*). Simplified approaches of the transfer functions such as the unit hydrograph are easy-to-use and parsimonious with a low number of parameters. We compare the one-parameter exponential model usually used in *SIRD* epidemiologic model to the two-parameter physically based Hayami model solution of the diffusive wave equation. Applications were implemented on the recent Covid-19 pandemic.

The application on 24 countries enables us to compare the performances of the two models. The exponential model gives very good performances for modelling the relationship *I*–*D*, but fair performances for modelling *I*–*R* and the number of active cases. For *I*–*R*, the Hayami model improves significantly the performances with excellent performances for all variables. The Hayami model presents also the advantage that its parameters can be easily estimated from the analysis of the data distributions of *I*(*t*), *R*(*t*) and *D*(*t*).

The Hayami model is parsimonious with only two parameters which are useful simple describers to compare the temporal evolution of recoveries and deaths in different countries with different contamination rates and strategies for recoveries.

These first results illustrate the interest of adapting mathematical formulations developed in a physical discipline like hydrology for applications in epidemiology. This allows epidemiology to benefit from the numerous advances in hydrology, and provides epidemiological modellers simple and easy-to-use parsimonious tools that have been evaluated in the literature, and could possibly make a modest contribution to the complex modelling exercise in epidemiology.

## Data Availability

Datasets for this research are available on the Worldometer's website https://www.worldometers.info/coronavirus/.

## Supplementary material

For supplementary material accompanying this paper visit http://dx.doi.org/10.1017/S0950268821001011. click here to view supplementary material
