# Supplementary material for: Use of the Hayami diffusive wave equation to model the relationship infected–recoveries–deaths of Covid-19 pandemic
Source: Epidemiol Infect. 2021 Apr 29;149:e138. doi: 10.1017/S0950268821001011 (PMC8207560; doi:10.1017/S0950268821001011)
Supplement: Supplementary file 1 [file S0950268821001011sup001.docx]

***Supplemental Material to***

**Use of the Hayami Diffusive Wave Equation**

**to Model the Relationship** **Infected-Recoveries-Deaths of Covid-19 Epidemic**

Roger Moussa^1*^ and Samer Majdalani^2^

^(1)^ LISAH, Univ. Montpellier, INRAE, IRD, Montpellier SupAgro, Montpellier, France.

^(2)^ HSM, CNRS, IRD, Univ. Montpellier, Montpellier, France.

**^*^** Corresponding author at: INRAE, UMR LISAH, 2 Place Pierre Viala, 34060 Montpellier, France

E-mail address: [roger.moussa@inrae.fr](mailto:roger.moussa@inrae.fr) (Roger Moussa)

The Supplemental Material presents the theory of the unit hydrograph, and then shows a comparison between the observed and calculated *R*(*t*), *D*(*t*) an *A*(*t*) for the 24 studied countries.

# The unit hydrograph theory

Note the input *I*(*t*) and the output *O*(*t*). On the basis of the unit hydrograph theory we have

$O\left( t \right)=\int_{0}^{t} I\left( t-\tau\right)u\left( \tau\right)d\tau=I\left( t \right)*u\left( t \right)$ (S1)

The symbol (*) represents the convolution relation. The function *u*(*t*) is called unit hydrograph, or a Kernel function, with

$\int_{0}^{\infty} u(t)dt=1$ (S2)

This property derives from the law of mass conservation (in hydrology the volume at the output equal to that on the input) with

$\int_{0}^{\infty} I(t)dt=\int_{0}^{\infty} O(t)dt$ (S3)

In epidemiology this property is verified because the total number of recovered corresponding to the total of *R*(*t*) is equal to the total of *I_R_*(*t*) = (1 – *μ*) *I*(*t*) (*μ* being the mortality ratio). The total number of deaths corresponding to the total of *D*(*t*) is also equal to the total of *I_D_*(*t*)= *μI*(*t*).

In the Hayami model we have

$u\left( t \right)=\left( \frac{\theta z}{\pi} \right)^{1/2} \frac{e^{\left[ z\left( 2-\frac{\theta}{t}-\frac{t}{\theta} \right) \right]}}{t^{3/2}}$ (S4)

with *θ* and *z* two parameters.

The Hayami *u*(*t*) given by equation (S4) is applicable for the continuous function data. But the Covid-19 pandemic problem dealing with *I-R-D* and active variables are observed in discrete time interval of *Δt =* 1 day herein. Hence *I* and *O* are discrete functions. Let *i* be an index representing the day and *I*(*i*) and *O*(*i*) the number of cases during the day *i*. Equation (S1) becomes:

$O\left( i \right)=\sum_{j=1}^{m} I\left( i-j+1 \right)a(j)$ (S5)

with *m* being the number of unit hydrograph ordinates, and *a*(*i*) the unit hydrograph ordinates for the time step *i* between [*t, t+Δt*]. We have (Figure S1)

$a\left( i \right)=\int_{t}^{t+\Delta t} u\left( \tau\right)d\tau$ (S6)

**Figure S1.** Term *a*(*i*) of a discrete unit hydrograph.

The integral of equation (S6) can be calculated either numerically using the trapezoid rule by choosing a time step of calculation *dτ* very small (for example *d = *1 minute in comparison to *Δt =* 1 day). For the Hayami equation (S4), Todini and Bossi (1986) gave an analytical solution (see the details in the Appendix of Todini and Bossi (1986), pp 417-424).

The property in equation (S2) becomes

$\sum_{i=1}^{m} a(i)=1$ (S7)

In the case of China (see Section 4.2), the calibrated parameters of the *I-R* Hayami model *u_R_*(*t*) are *θ_R_* = 21.8 days and* τ_R_* = 10.3 days (Figure 7a) with the corresponding *a_R_*(*i*) calculated using equation (S6)

*a_R_*(1) = 0.0000; *a_R_*(2) = 0.0000; *a_R_*(3) = 0.0000; *a_R_*(4) = 0.0000; *a_R_*(5) = 0.0004; *a_R_*(6) = 0.0021;

*a_R_*(7) = 0.0059; *a_R_*(8) = 0.0117; *a_R_*(9) = 0.0188; *a_R_*(10) = 0.0262; *a_R_*(11) = 0.0332; *a_R_*(12) = 0.0392;

*a_R_*(13) = 0.0438; *a_R_*(14) = 0.0470; *a_R_*(15) = 0.0489; *a_R_*(16) = 0.0497; *a_R_*(17) = 0.0494; *a_R_*(18) = .04830;

*a_R_*(19) = 0.0466; *a_R_*(20) = 0.0445; *a_R_*(21) = 0.0421; *a_R_*(22) = 0.0395; *a_R_*(23) = 0.0369; *a_R_*(24) = 0.0342;

*a_R_*(25) = 0.0315; *a_R_*(26) = 0.0290; *a_R_*(27) = 0.0265; *a_R_*(28) = 0.0242; *a_R_*(29) = 0.0220; *a_R_*(30) = 0.0200;

*a_R_*(31) = 0.0181; *a_R_*(32) = 0.0164; *a_R_*(33) = 0.0148; *a_R_*(34) = 0.0134; *a_R_* (35)= 0.0120; *a_R_*(36) = 0.0108;

etc.

The calibrated parameters of the *I-D* Hayami model *u_D_*(*t*) are *θ_D_* = 10.1 days and* τ_D_* = 11.1 days (Figure 7b) with the corresponding *a_D_*(*i*) calculated using equation (S6)

*a_D_*(1) = 0.0017; *a_D_*(2) = 0.0832; *a_D_*(3) = 0.1132; *a_D_*(4) = 0.1058; *a_D_*(5) = 0.0908; *a_D_*(6) = 0.0764;

*a_D_*(7) = 0.0642; *a_D_*(8) = 0.0541; *a_D_*(9) = 0.0460; *a_D_*(10) = 0.0393; *a_D_*(11) = 0.0339; *a_D_*(12) = 0.0294;

*a_D_*(13) = 0.0256; *a_D_*(14) = 0.0225; *a_D_*(15) = 0.0198; *a_D_*(16) = 0.0175; *a_D_*(17) = 0.0155; *a_D_*(18) = 0.0139;

*a_D_*(19) = 0.0124; *a_D_*(20) = 0.0111; *a_D_*(21) = 0.0100; *a_D_*(22) = 0.0090; *a_D_*(23) = 0.0082; *a_D_*(24) = 0.0074;

*a_D_*(25) = 0.0067; *a_D_*(26) = 0.0061; *a_D_*(27) = 0.0056; *a_D_*(28) = 0.0051; *a_D_*(29) = 0.0047; *a_D_*(30) = 0.0043;

*a_D_*(31) = 0.0039; *a_D_*(32) = 0.0036; *a_D_*(33) = 0.0033; *a_D_*(34) = 0.0030; *a_D_*(35) = 0.0028; *a_D_*(36) = 0.0026;

etc.

Figure S2 shows the discrete unit hydrographs *a_R_*(*i*) and *a_D_*(*i*).

**Figure S2.** The terms *a_R_*(*i*) and *a_D_*(*i*) of the discrete unit hydrographs respectively *u_R_*(*t*) and *u_D_*(*t*) in the case of China.

# Application on 24 countries

This section shows a comparison between the observed and calculated *R*(*t*), *D*(*t*) an *A*(*t*) for 24 countries (Figures S3 to S26): Australia (noted AU), Austria (AT), Belgium (BE), China (CN), Cuba (CU), Czechia (CZ), Denmark (DK), France (FR), Germany (DE), Iceland (IS), Iran (IR), Italy (IT), Japan (JP), Malaysia (MY), New Zealand (NZ), Romania (RO), Slovakia (SK), South Korea (KR), Spain (ES), Switzerland (CH), Thailand (TH), Turkey (TU), USA (US), and the whole world (World). Both the exponential and the Hayami models are compared using the calibrated parameters, for four smoothing levels of *I_o_*(*t*)*, R_o_*(*t*), and *D_o_*(*t*) (without smoothing, for 3-days moving average, for 5-days moving average, and for 7-days moving average), for the *NSE* performance criteria in Tables S1 (Exponential model) and S2 (Hayami model), and for the *KGE* performance criteria in Tables S3 (Exponential model) and S4 (Hayami model):

a) Comparison of the observed daily recoveries *R_o_*(*t*) and the calculated daily recoveries *R_c_*(*t*);

b) Comparison of the observed daily deaths *D_o_*(*t*) and the calculated daily deaths *D_c_*(*t*);

c) Comparison of he observed daily active cases *A_o_*(*t*) and the calculated daily active cases *A_c_*(*t*);

d) Comparison of the cumulated observed infected cases *I_ot_*(*t*), the observed cumulated number of recoveries *R_to_*(*t*)*,* the calculated cumulated number of recoveries *R_tc_*(*t*), the observed cumulated number of deaths *D_to_*(*t*), and the observed cumulated number of deaths *D_tc_*(*t*).

Data are available from 1/1 to 19/5/2020.

**List of Tables**

**Table S1.** The calibrated parameters and *NSE* performances criteria (for the *I-R* model, the *I-D* model, and the active cases) for the exponential model, for four smoothing levels of *I_o_*(*t*)*, R_o_*(*t*), and *D_o_*(*t*): *NSE_R_*, *NSE_D_*, and *NSE_A_* without smoothing; *NSE_R3_*, *NSE_D3_*, and *NSE_A3_* for 3-days moving average; *NSE_R5_*, *NSE_D5_*, and *NSE_A5_* for 5-days moving average; *NSE_R7_*, *NSE_D7_*, and *NSE_A7_* for 7-days moving average.

**Table S2.** The calibrated parameters and *NSE* performances criteria (for the *I-R* model, the *I-D* model, and the active cases) for the Hayami model, for four smoothing levels of *I_o_*(*t*)*, R_o_*(*t*), and *D_o_*(*t*): *NSE_R_*, *NSE_D_*, and *NSE_A_* without smoothing; *NSE_R3_*, *NSE_D3_*, and *NSE_A3_* for 3-days moving average; *NSE_R5_*, *NSE_D5_*, and *NSE_A5_* for 5-days moving average; *NSE_R7_*, *NSE_D7_*, and *NSE_A7_* for 7-days moving average.

**Table S3.** The calibrated parameters and *KGE* performances criteria (for the *I-R* model, the *I-D* model, and the active cases) for the exponential model, for four smoothing levels of *I_o_*(*t*)*, R_o_*(*t*), and *D_o_*(*t*): *KGE_R_*, *KGE_D_*, and *KGE_A_* without smoothing; *KGE_R3_*, *KGE_D3_*, and *KGE_A3_* for 3-days moving average; *KGE_R5_*, *KGE_D5_*, and *KGE_A5_* for 5-days moving average; *KGE_R7_*, *KGE_D7_*, and *KGE_A7_* for 7-days moving average.

**Table S4.** The calibrated parameters and *KGE* performances criteria (for the *I-R* model, the *I-D* model, and the active cases) for the Hayami model, for four smoothing levels of *I_o_*(*t*)*, R_o_*(*t*), and *D_o_*(*t*): *KGE_R_*, *KGE_D_*, and *KGE_A_* without smoothing; *KGE_R3_*, *KGE_D3_*, and *KGE_A3_* for 3-days moving average; *KGE_R5_*, *KGE_D5_*, and *KGE_A5_* for 5-days moving average; *KGE_R7_*, *KGE_D7_*, and *KGE_A7_* for 7-days moving average.

**Table S1.** The calibrated parameters and performances criteria (for the *I-R* model, the *I-D* model, and the active cases) for the exponential model, for four smoothing levels of *I_o_*(*t*)*, R_o_*(*t*), and *D_o_*(*t*): *NSE_R_*, *NSE_D_*, and *NSE_A_* without smoothing; *NSE_R3_*, *NSE_D3_*, and *NSE_A3_* for 3-days moving average; *NSE_R5_*, *NSE_D5_*, and *NSE_A5_* for 5-days moving average; *NSE_R7_*, *NSE_D7_*, and *NSE_A7_* for 7-days moving average.

| Country | *I-R* model | | | | |  | *I-D* model | | | | |  | Active cases | | | |
| --- | --- | --- | --- | --- | --- | --- | --- | --- | --- | --- | --- | --- | --- | --- | --- | --- |
|  | *k_R_* | *NSE_R_* | *NSE_R3_* | *NSE_R5_* | *NSE_R7_* |  | *k_D_* | *NSE_D_* | *NSE_D3_* | *NSE_D5_* | *NSE_D7_* |  | *NSE_A_* | *NSE_A3_* | *NSE_A5_* | *NSE_A7_* |
|  | days |  |  |  |  |  | (days) |  |  |  |  |  |  |  |  |  |
| Australia (AU) | 20.2 | 0.243 | 0.434 | 0.540 | 0.625 |  | 18.1 | 0.462 | 0.681 | 0.786 | 0.836 |  | 0.929 | 0.932 | 0.935 | 0.938 |
| Austria (AT) | 19.8 | 0.642 | 0.702 | 0.740 | 0.757 |  | 15.2 | 0.790 | 0.862 | 0.891 | 0.907 |  | 0.897 | 0.898 | 0.899 | 0.900 |
| Belgium (BE) | 8.5 | 0.705 | 0.810 | 0.845 | 0.865 |  | 5.5 | 0.787 | 0.877 | 0.901 | 0.915 |  | 0.670 | 0.669 | 0.667 | 0.666 |
| China (CN) | 26.3 | 0.660 | 0.675 | 0.682 | 0.691 |  | 9.3 | 0.936 | 0.966 | 0.977 | 0.984 |  | 0.860 | 0.860 | 0.859 | 0.859 |
| Cuba (CU) | 18.5 | 0.757 | 0.836 | 0.851 | 0.858 |  | 4.5 | 0.551 | 0.824 | 0.895 | 0.943 |  | 0.936 | 0.936 | 0.937 | 0.938 |
| Czechia (CZ) | 39.2 | 0.555 | 0.660 | 0.703 | 0.729 |  | 11.5 | 0.718 | 0.857 | 0.893 | 0.907 |  | 0.951 | 0.952 | 0.952 | 0.952 |
| Denmark (DK) | 15.1 | 0.488 | 0.671 | 0.749 | 0.799 |  | 1.5 | 0.767 | 0.854 | 0.884 | 0.900 |  | 0.882 | 0.884 | 0.886 | 0.889 |
| France (FR) | 24.3 | 0.738 | 0.818 | 0.877 | 0.891 |  | 6.1 | 0.934 | 0.971 | 0.983 | 0.990 |  | 0.976 | 0.976 | 0.976 | 0.976 |
| Germany (DE) | 16.1 | 0.701 | 0.841 | 0.912 | 0.939 |  | 16.1 | 0.793 | 0.878 | 0.921 | 0.938 |  | 0.960 | 0.961 | 0.962 | 0.962 |
| Iceland (IS) | 17.5 | 0.644 | 0.766 | 0.783 | 0.792 |  | 10.1 | 0.168 | 0.487 | 0.656 | 0.740 |  | 0.883 | 0.884 | 0.884 | 0.884 |
| Iran (IR) | 13.2 | 0.727 | 0.823 | 0.850 | 0.868 |  | 3.4 | 0.781 | 0.794 | 0.809 | 0.823 |  | 0.944 | 0.945 | 0.945 | 0.947 |
| Italy (IT) | 43.8 | 0.669 | 0.796 | 0.842 | 0.868 |  | 4.4 | 0.967 | 0.988 | 0.993 | 0.995 |  | 0.988 | 0.988 | 0.988 | 0.988 |
| Japan (JP) | 34.2 | 0.227 | 0.385 | 0.452 | 0.485 |  | 17.2 | 0.638 | 0.828 | 0.873 | 0.889 |  | 0.921 | 0.923 | 0.924 | 0.924 |
| Malaysia (MY) | 18.6 | 0.805 | 0.890 | 0.906 | 0.915 |  | 2.8 | 0.590 | 0.813 | 0.860 | 0.887 |  | 0.965 | 0.965 | 0.965 | 0.965 |
| New Zealand (NZ) | 21.5 | 0.633 | 0.736 | 0.765 | 0.782 |  | 20.5 | 0.203 | 0.410 | 0.463 | 0.517 |  | 0.885 | 0.886 | 0.886 | 0.887 |
| Romania (RO) | 26.5 | 0.591 | 0.779 | 0.826 | 0.862 |  | 6.7 | 0.888 | 0.965 | 0.978 | 0.984 |  | 0.961 | 0.961 | 0.962 | 0.962 |
| Slovakia (SK) | 29.5 | 0.379 | 0.594 | 0.665 | 0.689 |  | 10.7 | 0.316 | 0.537 | 0.633 | 0.688 |  | 0.926 | 0.927 | 0.928 | 0.929 |
| South Korea (KR) | 35.9 | 0.494 | 0.558 | 0.579 | 0.595 |  | 26.8 | 0.572 | 0.745 | 0.780 | 0.799 |  | 0.903 | 0.904 | 0.904 | 0.904 |
| Spain (ES) | 22.4 | 0.577 | 0.771 | 0.845 | 0.882 |  | 1.9 | 0.945 | 0.961 | 0.967 | 0.970 |  | 0.988 | 0.988 | 0.989 | 0.990 |
| Switzerland (CH) | 17.1 | 0.538 | 0.777 | 0.827 | 0.844 |  | 11.9 | 0.838 | 0.925 | 0.941 | 0.948 |  | 0.921 | 0.922 | 0.923 | 0.924 |
| Thailand (TH) | 16.2 | 0.505 | 0.677 | 0.712 | 0.742 |  | 9.1 | 0.602 | 0.805 | 0.850 | 0.874 |  | 0.886 | 0.887 | 0.889 | 0.891 |
| Turkey (TR) | 21.6 | 0.711 | 0.719 | 0.728 | 0.738 |  | 4.2 | 0.978 | 0.983 | 0.986 | 0.989 |  | 0.917 | 0.917 | 0.918 | 0.920 |
| USA (US) | 99.2 | 0.581 | 0.869 | 0.917 | 0.942 |  | 6.1 | 0.896 | 0.936 | 0.964 | 0.973 |  | 0.999 | 0.999 | 0.999 | 0.999 |
| World | 481 | 0.929 | 0.984 | 0.991 | 0.994 |  | 2.4 | 0.901 | 0.929 | 0.946 | 0.952 |  | 1.000 | 1.000 | 0.999 | 0.999 |

**Table S2.** The calibrated parameters and performances criteria (for the *I-R* model, the *I-D* model, and the active cases) for the Hayami model, for four smoothing levels of *I_o_*(*t*)*, R_o_*(*t*), and *D_o_*(*t*): *NSE_R_*, *NSE_D_*, and *NSE_A_* without smoothing; *NSE_R3_*, *NSE_D3_*, and *NSE_A3_* for 3-days moving average; *NSE_R5_*, *NSE_D5_*, and *NSE_A5_* for 5-days moving average; *NSE_R7_*, *NSE_D7_*, and *NSE_A7_* for 7-days moving average.

| Country | *I-R* model | | | | | |  | *I-D* model | | | | | |  | Active cases | | | |
| --- | --- | --- | --- | --- | --- | --- | --- | --- | --- | --- | --- | --- | --- | --- | --- | --- | --- | --- |
|  | *θ_R_* | *τ_R_* | *NSE_R_* | *NSE_R3_* | *NSE_R5_* | *NSE_R7_* |  | *θ_D_* | *τ_D_* | *NSE_D_* | *NSE_D3_* | *NSE_D5_* | *NSE_D7_* |  | *NSE_A_* | *NSE_A3_* | *NSE_A5_* | *NSE_A7_* |
|  | days | (days) |  |  |  |  |  | days | (days) |  |  |  |  |  |  |  |  |  |
| Australia (AU) |  | 9.5 | 0.309 | 0.554 | 0.680 | 0.770 |  |  | 14.5 | 0.482 | 0.712 | 0.818 | 0.865 |  | 0.964 | 0.967 | 0.969 | 0.972 |
| Austria (AT) | 16.5 | 5.5 | 0.850 | 0.926 | 0.973 | 0.987 |  | 15.0 | 9.0 | 0.844 | 0.923 | 0.953 | 0.971 |  | 0.994 | 0.995 | 0.995 | 0.995 |
| Belgium (BE) | 29 | 87 | 0.780 | 0.897 | 0.937 | 0.960 |  | 6.3 | 2 | 0.801 | 0.890 | 0.912 | 0.929 |  | 0.965 | 0.965 | 0.965 | 0.965 |
| China (CN) | 21.8 | 10.3 | 0.966 | 0.984 | 0.988 | 0.992 |  | 10.1 | 11.1 | 0.915 | 0.953 | 0.968 | 0.978 |  | 0.997 | 0.997 | 0.998 | 0.998 |
| Cuba (CU) | 18.5 | 4.5 | 0.872 | 0.966 | 0.977 | 0.983 |  | 5.5 | 8 | 0.543 | 0.815 | 0.888 | 0.936 |  | 0.990 | 0.990 | 0.991 | 0.991 |
| Czechia (CZ) |  | 14 | 0.751 | 0.892 | 0.952 | 0.978 |  |  | 8 | 0.756 | 0.897 | 0.931 | 0.944 |  | 0.995 | 0.995 | 0.995 | 0.995 |
| Denmark (DK) |  | 3.5 | 0.789 | 0.761 | 0.843 | 0.891 |  |  | 7.1 | 0.555 | 0.869 | 0.907 | 0.922 |  | 0.973 | 0.975 | 0.977 | 0.978 |
| France (FR) | 37 | 82 | 0.792 | 0.883 | 0.948 | 0.972 |  | 6.2 | 4.7 | 0.915 | 0.965 | 0.984 | 0.993 |  | 0.966 | 0.966 | 0.966 | 0.966 |
| Germany (DE) | 15 | 9 | 0.742 | 0.887 | 0.958 | 0.984 |  | 15.0 | 7.0 | 0.843 | 0.932 | 0.974 | 0.991 |  | 0.997 | 0.998 | 0.998 | 0.998 |
| Iceland (IS) | 16.5 | 6.0 | 0.817 | 0.965 | 0.983 | 0.991 |  | 11.5 | 5.5 | 0.164 | 0.492 | 0.673 | 0.768 |  | 0.997 | 0.998 | 0.998 | 0.998 |
| Iran (IR) | 11.3 | 1 | 0.823 | 0.928 | 0.951 | 0.961 |  | 5.5 | 8.0 | 0.772 | 0.784 | 0.799 | 0.812 |  | 0.974 | 0.974 | 0.973 | 0.973 |
| Italy (IT) | 43 | 31 | 0.736 | 0.875 | 0.924 | 0.949 |  | 4.4 | 2.7 | 0.964 | 0.987 | 0.994 | 0.997 |  | 0.988 | 0.988 | 0.988 | 0.988 |
| Japan (JP) | 28 | 2.5 | 0.438 | 0.740 | 0.866 | 0.946 |  | 16.5 | 10 | 0.666 | 0.869 | 0.915 | 0.936 |  | 0.955 | 0.996 | 0.997 | 0.997 |
| Malaysia (MY) | 16.5 | 10 | 0.828 | 0.914 | 0.931 | 0.941 |  | 4.0 | 2.5 | 0.578 | 0.809 | 0.859 | 0.887 |  | 0.957 | 0.957 | 0.957 | 0.956 |
| New Zealand (NZ) | 15.5 | 6.5 | 0.788 | 0.914 | 0.946 | 0.958 |  | 18.5 | 1 | 0.388 | 0.798 | 0.860 | 0.911 |  | 0.990 | 0.991 | 0.991 | 0.991 |
| Romania (RO) | 26.0 | 8.5 | 0.651 | 0.862 | 0.904 | 0.940 |  | 6.1 | 8 | 0.886 | 0.962 | 0.977 | 0.983 |  | 0.990 | 0.990 | 0.991 | 0.992 |
| Slovakia (SK) | 23.5 | 6.5 | 0.532 | 0.828 | 0.921 | 0.941 |  | 10.5 | 2 | 0.349 | 0.584 | 0.684 | 0.732 |  | 0.988 | 0.989 | 0.989 | 0.989 |
| South Korea (KR) | 25.3 | 9.8 | 0.835 | 0.939 | 0.962 | 0.972 |  | 30.2 | 27.0 | 0.524 | 0.684 | 0.723 | 0.749 |  | 0.969 | 0.969 | 0.969 | 0.969 |
| Spain (ES) | 23.8 | 24.4 | 0.563 | 0.758 | 0.834 | 0.873 |  | 2.8 | 3.0 | 0.950 | 0.966 | 0.973 | 0.976 |  | 0.991 | 0.992 | 0.993 | 0.993 |
| Switzerland (CH) | 14.4 | 1.6 | 0.638 | 0.912 | 0.956 | 0.965 |  | 11.0 | 6.0 | 0.880 | 0.969 | 0.984 | 0.990 |  | 0.972 | 0.972 | 0.972 | 0.972 |
| Thailand (TH) | 16.3 | 7.2 | 0.585 | 0.784 | 0.824 | 0.857 |  | 9.7 | 2.1 | 0.661 | 0.888 | 0.939 | 0.967 |  | 0.967 | 0.968 | 0.970 | 0.971 |
| Turkey (TR) | 19.6 | 1.3 | 0.950 | 0.960 | 0.967 | 0.972 |  | 5.5 | 8.8 | 0.979 | 0.984 | 0.988 | 0.991 |  | 0.998 | 0.998 | 0.998 | 0.998 |
| USA (US) | 89 | 99 | 0.572 | 0.851 | 0.901 | 0.921 |  | 6.5 | 2.7 | 0.976 | 0.953 | 0.975 | 0.981 |  | 0.999 | 0.999 | 0.999 | 0.999 |
| World | 47.5 | 47.5 | 0.917 | 0.971 | 0.978 | 0.980 |  | 5.9 | 9 | 0.888 | 0.915 | 0.932 | 0.937 |  | 0.994 | 0.994 | 0.994 | 0.994 |

**Table S3.** The calibrated parameters and *KGE* performances criteria (for the *I-R* model, the *I-D* model, and the active cases) for the exponential model, for four smoothing levels of *I_o_*(*t*)*, R_o_*(*t*), and *D_o_*(*t*): *KGE_R_*, *KGE_D_*, and *KGE_A_* without smoothing; *KGE_R3_*, *KGE_D3_*, and *KGE_A3_* for 3-days moving average; *KGE_R5_*, *KGE_D5_*, and *KGE_A5_* for 5-days moving average; *KGE_R7_*, *KGE_D7_*, and *KGE_A7_* for 7-days moving average.

| Country | *I-R* model | | | | |  | *I-D* model | | | | |  | Active cases | | | |
| --- | --- | --- | --- | --- | --- | --- | --- | --- | --- | --- | --- | --- | --- | --- | --- | --- |
|  | *k_R_* | *KGE_R_* | *KGE_R3_* | *KGE_R5_* | *KGE_R7_* |  | *k_D_* | *KGE_D_* | *KGE_D3_* | *KGE_D5_* | *KGE_D7_* |  | *KGE_A_* | *KGE_A3_* | *KGE_A5_* | *KGE_A7_* |
|  | days |  |  |  |  |  | (days) |  |  |  |  |  |  |  |  |  |
| Australia (AU) | 20.2 | 0.240 | 0.456 | 0.553 | 0.618 |  | 18.1 | 0.517 | 0.711 | 0.790 | 0.822 |  | 0.849 | 0.852 | 0.854 | 0.857 |
| Austria (AT) | 19.8 | 0.607 | 0.650 | 0.675 | 0.686 |  | 15.2 | 0.750 | 0.796 | 0.813 | 0.821 |  | 0.856 | 0.857 | 0.858 | 0.860 |
| Belgium (BE) | 8.5 | 0.711 | 0.692 | 0.673 | 0.666 |  | 5.5 | 0.764 | 0.821 | 0.835 | 0.843 |  | 0.484 | 0.484 | 0.482 | 0.481 |
| China (CN) | 26.3 | 0.603 | 0.611 | 0.615 | 0.619 |  | 9.3 | 0.966 | 0.982 | 0.985 | 0.987 |  | 0.739 | 0.739 | 0.739 | 0.740 |
| Cuba (CU) | 18.5 | 0.679 | 0.721 | 0.737 | 0.742 |  | 4.5 | 0.618 | 0.843 | 0.893 | 0.922 |  | 0.826 | 0.826 | 0.826 | 0.827 |
| Czechia (CZ) | 39.2 | 0.543 | 0.628 | 0.649 | 0.672 |  | 11.5 | 0.699 | 0.789 | 0.810 | 0.819 |  | 0.820 | 0.820 | 0.820 | 0.820 |
| Denmark (DK) | 15.1 | 0.502 | 0.654 | 0.710 | 0.742 |  | 1.5 | 0.756 | 0.811 | 0.820 | 0.822 |  | 0.812 | 0.813 | 0.814 | 0.814 |
| France (FR) | 24.3 | 0.818 | 0.839 | 0.836 | 0.822 |  | 6.1 | 0.917 | 0.941 | 0.949 | 0.952 |  | 0.962 | 0.0962 | 0.962 | 0.962 |
| Germany (DE) | 16.1 | 0.702 | 0.801 | 0.844 | 0.859 |  | 16.1 | 0.768 | 0.824 | 0.847 | 0.857 |  | 0.910 | 0.911 | 0.911 | 0.912 |
| Iceland (IS) | 17.5 | 0.609 | 0.691 | 0.702 | 0.707 |  | 10.1 | 0.129 | 0.516 | 0.664 | 0.730 |  | 0.792 | 0.792 | 0.793 | 0.795 |
| Iran (IR) | 13.2 | 0.744 | 0.815 | 0.834 | 0.846 |  | 3.4 | 0.872 | 0.880 | 0.885 | 0.889 |  | 0.905 | 0.905 | 0.905 | 0.905 |
| Italy (IT) | 43.8 | 0.673 | 0.764 | 0.794 | 0.814 |  | 4.4 | 0.969 | 0.983 | 0.985 | 0.986 |  | 0.920 | 0.920 | 0.920 | 0.920 |
| Japan (JP) | 34.2 | 0.177 | 0.357 | 0.419 | 0.433 |  | 17.2 | 0.608 | 0.725 | 0.754 | 0.757 |  | 0.767 | 0.767 | 0.768 | 0.770 |
| Malaysia (MY) | 18.6 | 0.804 | 0.862 | 0.873 | 0.880 |  | 2.8 | 0.595 | 0.760 | 0.788 | 0.801 |  | 0.834 | 0.834 | 0.834 | 0.834 |
| New Zealand (NZ) | 21.5 | 0.586 | 0.656 | 0.674 | 0.684 |  | 20.5 | 0.155 | 0.391 | 0.438 | 0.483 |  | 0.824 | 0.824 | 0.825 | 0.826 |
| Romania (RO) | 26.5 | 0.605 | 0.745 | 0.788 | 0.815 |  | 6.7 | 0.889 | 0.934 | 0.941 | 0.946 |  | 0.807 | 0.807 | 0.806 | 0.806 |
| Slovakia (SK) | 29.5 | 0.360 | 0.546 | 0.592 | 0.610 |  | 10.7 | 0.313 | 0.525 | 0.601 | 0.641 |  | 0.798 | 0.798 | 0.798 | 0.799 |
| South Korea (KR) | 35.9 | 0.501 | 0.557 | 0.572 | 0.582 |  | 26.8 | 0.624 | 0.764 | 0.789 | 0.800 |  | 0.868 | 0.868 | 0.869 | 0.870 |
| Spain (ES) | 22.4 | 0.634 | 0.798 | 0.858 | 0.883 |  | 1.9 | 0.878 | 0.883 | 0.884 | 0.884 |  | 0.962 | 0.962 | 0.962 | 0.962 |
| Switzerland (CH) | 17.1 | 0.540 | 0.721 | 0.753 | 0.763 |  | 11.9 | 0.800 | 0.853 | 0.861 | 0.864 |  | 0.848 | 0.848 | 0.849 | 0.850 |
| Thailand (TH) | 16.2 | 0.515 | 0.656 | 0.682 | 0.702 |  | 9.1 | 0.609 | 0.762 | 0.792 | 0.808 |  | 0.847 | 0.848 | 0.850 | 0.852 |
| Turkey (TR) | 21.6 | 0.663 | 0.669 | 0.675 | 0.681 |  | 4.2 | 0.917 | 0.918 | 0.919 | 0.918 |  | 0.785 | 0.785 | 0.785 | 0.785 |
| USA (US) | 99.2 | 0.655 | 0.891 | 0.920 | 0.936 |  | 6.1 | 0.858 | 0.878 | 0.890 | 0.897 |  | 0.976 | 0.976 | 0.976 | 0.976 |
| World | 481 | 0.959 | 0.962 | 0.961 | 0.961 |  | 2.4 | 0.931 | 0.947 | 0.955 | 0.958 |  | 0.987 | 0.987 | 0.986 | 0.986 |

**Table S4.** The calibrated parameters and *KGE* performances criteria (for the *I-R* model, the *I-D* model, and the active cases) for the Hayami model, for four smoothing levels of *I_o_*(*t*)*, R_o_*(*t*), and *D_o_*(*t*): *KGE_R_*, *KGE_D_*, and *KGE_A_* without smoothing; *KGE_R3_*, *KGE_D3_*, and *KGE_A3_* for 3-days moving average; *KGE_R5_*, *KGE_D5_*, and *KGE_A5_* for 5-days moving average; *KGE_R7_*, *KGE_D7_*, and *KGE_A7_* for 7-days moving average.

| Country | *I-R* model | | | | | |  | *I-D* model | | | | | |  | Active cases | | | |
| --- | --- | --- | --- | --- | --- | --- | --- | --- | --- | --- | --- | --- | --- | --- | --- | --- | --- | --- |
|  | *θ_R_* | *τ_R_* | *KGE_R_* | *KGE_R3_* | *KGE_R5_* | *KGE_R7_* |  | *θ_D_* | *τ_D_* | *KGE_D_* | *KGE_D3_* | *KGE_D5_* | *KGE_D7_* |  | *KGE_A_* | *KGE_A3_* | *KGE_A5_* | *KGE_A7_* |
|  | days | (days) |  |  |  |  |  | days | (days) |  |  |  |  |  |  |  |  |  |
| Australia (AU) |  | 9.5 | 0.362 | 0.625 | 0.739 | 0.814 |  |  | 14.5 | 0.585 | 0.797 | 0.876 | 0.907 |  | 0.831 | 0.832 | 0.833 | 0.834 |
| Austria (AT) | 16.5 | 5.5 | 0.887 | 0.943 | 0.974 | 0.980 |  | 15.0 | 9.0 | 0.870 | 0.926 | 0.945 | 0.955 |  | 0.931 | 0.931 | 0.931 | 0.931 |
| Belgium (BE) | 29 | 87 | 0.844 | 0.894 | 0.890 | 0.889 |  | 6.3 | 2 | 0.797 | 0.857 | 0.870 | 0.878 |  | 0.848 | 0.848 | 0.846 | 0.846 |
| China (CN) | 21.8 | 10.3 | 0.979 | 0.987 | 0.988 | 0.988 |  | 10.1 | 11.1 | 0.957 | 0.969 | 0.973 | 0.976 |  | 0.945 | 0.945 | 0.946 | 0.946 |
| Cuba (CU) | 18.5 | 4.5 | 0.872 | 0.922 | 0.940 | 0.943 |  | 5.5 | 8 | 0.620 | 0.844 | 0.892 | 0.920 |  | 0.909 | 0.908 | 0.909 | 0.910 |
| Czechia (CZ) |  | 14 | 0.815 | 0.910 | 0.946 | 0.939 |  |  | 8 | 0.756 | 0.851 | 0.873 | 0.881 |  | 0.936 | 0.935 | 0.935 | 0.935 |
| Denmark (DK) |  | 3.5 | 0.610 | 0.782 | 0.845 | 0.879 |  |  | 7.1 | 0.807 | 0.868 | 0.879 | 0.885 |  | 0.969 | 0.969 | 0.969 | 0.969 |
| France (FR) | 37 | 82 | 0.850 | 0.872 | 0.865 | 0.850 |  | 6.2 | 4.7 | 0.932 | 0.963 | 0.972 | 0.976 |  | 0.829 | 0.829 | 0.829 | 0.828 |
| Germany (DE) | 15 | 9 | 0.800 | 0.914 | 0.966 | 0.985 |  | 15.0 | 7.0 | 0.901 | 0.963 | 0.979 | 0.974 |  | 0.960 | 0.960 | 0.960 | 0.960 |
| Iceland (IS) | 16.5 | 6.0 | 0.834 | 0.938 | 0.948 | 0.952 |  | 11.5 | 5.5 | 0.153 | 0.570 | 0.731 | 0.803 |  | 0.964 | 0.964 | 0.964 | 0.964 |
| Iran (IR) | 11.3 | 1 | 0.868 | 0.944 | 0.961 | 0.967 |  | 5.5 | 8.0 | 0.875 | 0.882 | 0.889 | 0.894 |  | 0.862 | 0.862 | 0.860 | 0.858 |
| Italy (IT) | 43 | 31 | 0.773 | 0.864 | 0.889 | 0.904 |  | 4.4 | 2.7 | 0.978 | 0.987 | 0.986 | 0.986 |  | 0.892 | 0.892 | 0.892 | 0.892 |
| Japan (JP) | 28 | 2.5 | 0.493 | 0.769 | 0.862 | 0.875 |  | 16.5 | 10 | 0.699 | 0.830 | 0.861 | 0.862 |  | 0.963 | 0.964 | 0.965 | 0.965 |
| Malaysia (MY) | 16.5 | 10 | 0.877 | 0.914 | 0.916 | 0.915 |  | 4.0 | 2.5 | 0.591 | 0.762 | 0.790 | 0.804 |  | 0.823 | 0.823 | 0.822 | 0.822 |
| New Zealand (NZ) | 15.5 | 6.5 | 0.866 | 0.956 | 0.965 | 0.964 |  | 18.5 | 1 | 0.452 | 0.816 | 0.869 | 0.915 |  | 0.947 | 0.947 | 0.947 | 0.947 |
| Romania (RO) | 26.0 | 8.5 | 0.702 | 0.850 | 0.897 | 0.922 |  | 6.1 | 8 | 0.893 | 0.940 | 0.948 | 0.952 |  | 0.900 | 0.900 | 0.901 | 0.903 |
| Slovakia (SK) | 23.5 | 6.5 | 0.612 | 0.859 | 0.920 | 0.927 |  | 10.5 | 2 | 0.396 | 0.630 | 0.710 | 0.746 |  | 0.897 | 0.897 | 0.897 | 0.897 |
| South Korea (KR) | 25.3 | 9.8 | 0.900 | 0.914 | 0.906 | 0.901 |  | 30.2 | 27.0 | 0.640 | 0.780 | 0.807 | 0.822 |  | 0.882 | 0.882 | 0.882 | 0.882 |
| Spain (ES) | 23.8 | 24.4 | 0.648 | 0.818 | 0.880 | 0.908 |  | 2.8 | 3.0 | 0.919 | 0.925 | 0.926 | 0.927 |  | 0.992 | 0.993 | 0.993 | 0.994 |
| Switzerland (CH) | 14.4 | 1.6 | 0.754 | 0.946 | 0.937 | 0.932 |  | 11.0 | 6.0 | 0.908 | 0.971 | 0.979 | 0.982 |  | 0.857 | 0.857 | 0.857 | 0.857 |
| Thailand (TH) | 16.3 | 7.2 | 0.651 | 0.818 | 0.848 | 0.872 |  | 9.7 | 2.1 | 0.728 | 0.907 | 0.942 | 0.959 |  | 0.875 | 0874 | 0.874 | 0.874 |
| Turkey (TR) | 19.6 | 1.3 | 0.951 | 0.955 | 0.959 | 0.961 |  | 5.5 | 8.8 | 0.969 | 0.971 | 0.972 | 0.972 |  | 0.985 | 0.985 | 0.985 | 0.985 |
| USA (US) | 89 | 99 | 0.700 | 0.924 | 0.939 | 0.932 |  | 6.5 | 2.7 | 0.881 | 0.900 | 0.912 | 0.920 |  | 0.958 | 0.058 | 0.959 | 0.960 |
| World | 47.5 | 47.5 | 0.935 | 0.937 | 0.940 | 0.941 |  | 5.9 | 9 | 0.932 | 0.950 | 0.959 | 0.962 |  | 0.921 | 0.921 | 0.922 | 0.924 |

**Figure S3.** Australia (AU).

**Figure S4.** Austria (AT).

**Figure S5.** Belgium (BE).

**Figure S6.** China (CN).

**Figure S7.** Cuba (CU).

**Figure S8.** Czechia (CZ).

**Figure S9.** Denmark (DK).

**Figure S10.** France (FR).

**Figure S11.** Germany (DE).

**Figure S12.** Iceland (IS).

**Figure S13.** Iran (IR).

**Figure S14.** Italy (IT).

**Figure S15.** Japan (JP).

**Figure S16.** Malaysia (MY).

**Figure S17.** New Zealand (NZ).

**Figure S18.** Romania (RO).

**Figure S19.** Slovakia (SK).

**Figure S20** South Korea (KR).

**Figure S21.** Spain (ES).

**Figure S22.** Switzerland (CH).

**Figure S23.** Thailand (TH).

**Figure S24.** Turkey (TU).

**Figure S25.** USA (US).

**Figure S26.** The world.
